# Compounds with a ‘stuffed’ anti-bixbyite-type structure, analysed in terms of the Zintl–Klemm and coordination-defect concepts

**DOI:** 10.1107/S010876810803423X

**Published:** 2008-12-20

**Authors:** Angel Vegas, Raymond L. Martin, D. J. M. Bevan

**Affiliations:** a Instituto de Química Física ‘Rocasolano’, CSIC, C/Serrano 119, 28006 Madrid, Spain; b Monash University, School of Chemistry, Clayton, Victoria, Australia; c The Flinders University of South Australia, School of Chemistry, Physics and Earth Sciences, Bedford Park, South Australia

**Keywords:** Zintl–Klemm concept, coordination-defect concept, multiple resonance structure, electron transfer

## Abstract

The bixbyite structure (Mn_2_O_3_) () is often described as a distorted face-centered cubic (f.c.c.) array of Mn atoms, with O atoms occupying 3/4 of the tetrahedral holes. The empty *M*_4_ tetrahedra are centred at 16*c*. In anti-bixbyite structures (Mg_3_N_2_), cation vacancies are centred in empty N_4_ tetrahedra. If 16 hypothetical atoms were located at this site they would form the structure of γ-Si. This means that anti-bixbyite structures are ideally prepared to accommodate Si(Ge) atoms at these holes. Several compounds (Li_3_AlN_2_ and Li_3_ScN_2_) fully satisfy this expectation. They are really anti-bixbyites ‘stuffed’ with Al(Sc). The presence of these atoms in 16*c* is illuminated in the light of the extended Zintl–Klemm concept (EZKC) [Vegas & García-Baonza (2007 ▶). *Acta Cryst.* B**63**, 339–345], from which a compound would be the result of ‘multiple resonance’ pseudo-structures, emerging from electron transfers between any species pair (like or unlike atoms, cations or anions). The coordination-defect (CD) concept [Bevan & Martin (2008) ▶. *J. Solid State Chem.* **181**, 2250–2259] is also consistent with the EZKC description of the pseudo-structures. A more profound insight into crystal structures is gained if one is not restricted to the contemplation of classical anions and cations in their conventional oxidation states.

## Abstract 2

Compounds with a ‘stuffed anti-bixbyite’ structure, such as Li_3_AlN_2_, were analysed in terms of both the extended Zintl–Klemm concept and the coordination-defect concept. For the first time, inorganic crystal structures are seen as a set of ‘multiple resonance structures’ (Klemm pseudo-structures) which co-exist as the result of unexpected electron transfers between any species pair comprising either like or unlike atoms, cations or anions. If this is the driving force controlling crystal structures, the conventional oxidation states assigned to cations and anions lose some of their usefulness.

## 1. Introduction

The mineral bixbyite (Mn_2_O_3_) and isostructural compounds like Sc_2_O_3_ and In_2_O_3_ (Zachariasen, 1928 ▶) are cubic. The space group of bixbyite is 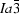, with *Z* = 16. The Mn atoms occupy two crystallographically independent sites, 8*b* and 24*d*, whereas all the O atoms are equivalent, located at 48*e*. The structure has often been described (Wells, 1975 ▶) as a slightly distorted f.c.c. array of Mn atoms, with the O atoms occupying ¾ of the tetrahedral holes. Hence, bixbyite has also been regarded as an anion-deficient, fluorite-related structure, M_4_O_6_□_2_, with the vacant site, designated □, located in the anion sublattice.

There are well known anti-bixbyite-type compounds such as N_2_Mg_3_ (von Stackelberg & Paulus, 1933 ▶): this example can be similarly regarded as a cation-deficient, anti-fluorite structure, N_2_Mg_3_□_*M*_, with the vacant site, □_*M*_, now located in the cation sublattice.

The similarity (topology and bond lengths) of cation arrays with the structures of the parent metals was also reported by Ramos-Gallardo & Vegas (1995 ▶), as an example of what have been called ‘real stuffed alloys’ (Martínez-Cruz *et al.*, 1994 ▶; Vegas, 2000 ▶; Vegas & Jansen, 2002 ▶; Vegas *et al.*, 2001 ▶). Very recently, Bevan & Martin (2008 ▶) have reported a crystal-chemical study in which their ‘coordination-defect theory’ of anion vacancies or voids was applied to analyse the structures of the anion-deficient, fluorite-related sesquioxide minerals bixbyite, braunite and parwelite.

**Figure 1:**
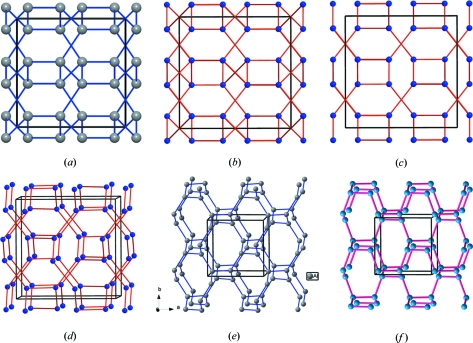
(*a*) A projection of the cubic structure of the metastable HP phase γ-Si(Ge). (*b*) The substructure of the fictitious V atoms (voids), occupying the 16*c* site in the bixbyite structure Mn_2_O_3_. (*c*) The same drawing as (*b*) in which some contacts (bonds) have been eliminated to show clearly how the structure can be decomposed into two three-connected subsets. (*d*) Perspective view of (*c*) to emphasize the threefold connection in each subset, which is characteristic of the Group 15 elements (N, P). Note the existence of both tetragonal and octagonal helices, perpendicular to the projection plane. (*e*) The Al sub-array in Sr_3_Al_2_O_5_Cl_2_, where the Al atoms are converted into (Ψ-P), forming a (Ψ-P_2_O_5_) skeleton. The network is identical to the subsets forming both the HP-Si(Ge) and the V voids in bixbyite. (*f*) The structure of the high-pressure, high-temperature N to show its similarity with both the Al subarray represented in (*e*) and the two subsets of HP-Si, represented in (*d*).

In that paper, Bevan & Martin (2008 ▶) made an unusual observation, *i.e.* they noticed that in bixbyite the 16 vacancies (a quarter of the tetrahedral holes in the f.c.c. cation array) correspond to the positions of the 16*c* site of the space group 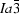, and that when an anion vacancy, □, is located at this site, the pattern represented in Fig. 1 ▶(*b*) is obtained. Looking at this array, we soon recognized that the pattern is coincident with that of the quenched high-pressure γ phases of Si and Ge (Wentorf & Kasper, 1963 ▶; Kasper & Richards, 1964 ▶; see Fig. 1 ▶ *a*): these have not only the same space group (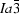) as bixbyite, but the Si(Ge) atoms occupy the same 16*c* sites as the bixbyite vacancies. Both structures are compared in Figs. 1 ▶(*a*) and (*b*).

At first glance, this feature could be considered as an accidental and unremarkable coincidence. However, encouraged by our intuition, and taking into account that many structures can be illuminated by the Zintl–Klemm concept (hereafter ZKC; Zintl, 1939 ▶; Klemm, 1958 ▶), we decided that this structural coincidence was worthy of a deeper analysis. This decision was based on two observed features. On the one hand, there was our previous experience of how the elemental structures were often preserved in the compounds (Vegas, 2000 ▶; Vegas & Jansen, 2002 ▶), and on the other because the presence of ‘foreign’ atoms can stabilize a given structure, as happens in the Zintl phases (Santamaría-Pérez & Vegas, 2003 ▶; Santamaría-Pérez *et al.*, 2005 ▶; Vegas & García-Baonza, 2007 ▶). This feature occurs not only in alloys but also in oxides and can be considered as an extension of the Zintl–Klemm concept. Our first thought was that tetrahedral voids, □_*M*_, in the cation sublattice of the anti-bixbyite structure should be the ideal sites to accommodate an atom such as Si or Ge: in other words, it would seem that the anti-bixbyite structure ‘is well prepared to accommodate Si(Ge) atoms that, when present, should necessarily be located in these holes’.

To test this hypothesis, we have undertaken the present study to ascertain whether ‘stuffed’ anti-bixbyite-type compounds of the general stoichiometry *M*_3_Si*X*_2_, could exist, and also whether the Si (or indeed other) atoms would be located at the predicted positions.

Subsequently, we discovered the prior existence of a paper by Niewa *et al.* (2003 ▶) on the structure of Li_3_ScN_2_, in which they reported thoughts very similar to ours. Their paper contains the following quote: ‘Focussing on the [Sc(N^3−^)_4/2_] framework, the Sc arrangement is topologically equivalent to the Si arrangement in (high-pressure, high-density) γ-Si … As the Li_3_N_2_ substructure is isostructural with that of Mg_3_N_2_ and α-Ca_3_N_2_ (anti-bixbyite), the [Sc(N^3−^)_4/2_] framework consequently corresponds to the occupation of the unoccupied tetrahedral holes in the anti-bixbyite structure with Sc’. **However, Sc is not Si(Ge)!** The electron configuration and stereochemical preferences of Sc usually differ substantially from those of Si and Ge.

## 2. Results and discussion

### 2.1. Extended Zintl–Klemm concept

If our hypothesis is valid, the existence of some compounds of the general formula *M*_3_Si*X*_2_ would be expected. For such a compound, the sub-array *M*_3_ *X*_2_ should have the desired anti-bixbyite structure, and the foreign atom (Si, Ge) should be located at the 16*c* site of the space group 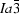. Considered as a whole, without any distinction between cations, the compound would be formulated as *M*_4_ *X*_2_, which corresponds to the stoichiometry of an anti-fluorite structure, *i.e.* *M*_2_ *X* (Mg_2_Si, for example; Owen & Preston, 1924 ▶), rather than that of fluorite.

We now know of the existence of numerous ternary compounds with the same space group 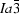, such as, for example, Li_3_AlN_2_, Li_3_GaN_2_, Li_3_[Ge_0.67_Li_0.33_]N_2_ and, more recently, another such compound, Li_3_ScN_2_, as quoted above. Then there are other compounds of the general formula Li_3_[(*M* ^IV^)_0.67_Li_0.33_]N_2_, with *M* ^IV^ = Si, Ti. In all of these, the cations of the [(*M* ^IV^)_0.67_Li_0.33_] group are disordered.

Furthermore, there is also a group of compounds, Li_7_ *M* ^V^N_4_ (*M* ^V^ = Mn, V, Nb, Ta), which crystallize in the space group 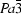, a sub-group of 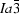 from which the body-centering operation has been lost. The formulae should more correctly be written as Li_6_[*M* ^V^Li]N_4_, by analogy with Li_3_[(*M* ^IV^)_0.67_Li_0.33_]N_2_ (*M* ^IV^ = Ge), since the Li and *M* ^V^ atoms in the square brackets are now ordered on two 8*c* sites of the space group, corresponding almost exactly with the 16*c* sites of 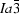. All these compounds, as well as their references are summarized in Table 1 ▶.

In these various compounds, it is encouraging to see that the Al(Ga,Sc) atoms, the disordered sets [(*M* ^IV^)_0.67_Li_0.33_] and the ordered sets [Li*M* ^V^] are all located in the relevant sites which give rise to the γ-Si framework. However, the crucial question is: why are the diverse atoms of these sets disposed in the tetrahedral network expected for Si(Ge)? The first impression might be that our assumption was not justified, and indeed this raises the possibility that any of the other atomic species could be lodged in these voids. However, this apparent contradiction can be resolved in the context of the extended Zintl–Klemm concept (Vegas & García-Baonza, 2007 ▶), as we shall demonstrate below. It is important to note that the application of the Zintl–Klemm concept to a compound invariably allows for several alternative structural interpretations. This concept and, especially, Klemm’s pseudo-atom concept, correlate the stereochemical properties of an atom with the transfer of electrons from a donor to an acceptor atom. Both atoms are thereby transposed to pseudo-atoms, each with a new electronic configuration and stereochemical properties (Santamaría-Pérez & Vegas, 2003 ▶; Santamaría-Pérez *et al.*, 2005 ▶; Vegas & García-Baonza, 2007 ▶). It is generally accepted that the pseudo-atoms are denoted by the prefix Ψ (See Table 2 ▶). For example, when an Al atom accepts one electron, it becomes isoelectronic with a Si pseudo-atom, designated as (Ψ-Si). These authors have also provided examples of the general application of the Zintl concept to explain the structure of several inorganic structures, and to demonstrate how many of them are stabilized by the presence of foreign atoms.

In the Li_3_Al(Ga,Sc)N_2_ structure (Juza & Hund, 1948 ▶; Niewa *et al.*, 2003 ▶) the Al(Ga,Sc) or Ψ-Si(Ge) atoms are four-connected, following the 8-*N* rule, as occurs with most structures of the *p*-block elements. The tetrahedral connection (characteristic of the Group 14 elements) is made in such a way that what are seen as octagons and squares in projection are really octagonal and square helices about 2_1_ axes perpendicular to the projection plane. See Figs. 1 ▶(*d*), (*e*) and (*f*). It is immediately evident that these skeletons resemble those of the Si sub-array in the quartz structure in which trigonal and hexagonal helices coexist.

In the structures of the γ-Si(Ge) phases the four linkages are almost equal (Si—Si distances of 3 × 2.38 Å, 1 × 2.37 Å), whereas in Li_3_AlN_2_, the equivalent Al—Al distances show a somewhat greater difference (3 × 3.61 Å, 1 × 3.85 Å): in the case of Li_3_ScN_2_, these distances are 3 × 3.55 and 1 × 4.25. If the single longer linkage in each case were to be omitted, we would obtain the pattern represented in Fig. 1 ▶(*c*) for Li_3_AlN_2_. This consists of two infinite, interpenetrating subsets. These independent, three-connected Al networks simply interpenetrate, as shown in Figs. 1 ▶(*c*) and (*d*).

It should be remarked that this three-connected network is the one expected for the Group 15 elements (N, P, As *etc.*), and has been identified as the Al sub-array in Sr_3_Al_2_O_5_Cl_2_ (Santamaría-Pérez, 2006 ▶). This skeleton is represented in Fig. 1 ▶(*e*) and its structure can be interpreted in light of the ZKC. Here, the three Sr atoms act as donors. One of them transfers two electrons to the two Cl atoms. The two remaining Sr atoms transfer their four valence electrons to the Al atoms, converting them into (Ψ-P) and, consequently, the Al_2_O_5_ group becomes (Ψ-P_2_O_5_). Compare Figs. 1 ▶(*d*) and (*e*).

The structural behaviour of molecular N_2_ illustrates this interpretation. At 115 GPa and 2600 K, nitrogen undergoes a phase transition adopting the three-connected structure represented in Fig. 1 ▶(*f*) (Eremets *et al.*, 2004 ▶). The structure is also cubic, *I*2_1_3, with *a* = 3.45 Å. The N atoms are located at 8*a* (*x*, *x*, *x*: *x* = 0.067), forming the same structure as the Al atoms (Ψ-P) in Sr_3_Al_2_O_5_Cl_2_ (Leib & Müller-Buschbaum, 1986 ▶). The important issue here is that the Al network, discovered in Sr_3_Al_2_O_5_Cl_2_ (Santamaría-Pérez & Vegas, 2003 ▶), is by no means a hypothetical structure for the Group 15 elements but it really exists as a stable phase for nitrogen! Compare Figs. 1 ▶(*e*) and 1(*f*). The similarity of this network with the Si skeleton (Ψ-P) in the Zintl phase SrSi_2_ (Pringle, 1972 ▶), is also remarkable.

**Figure 2:**
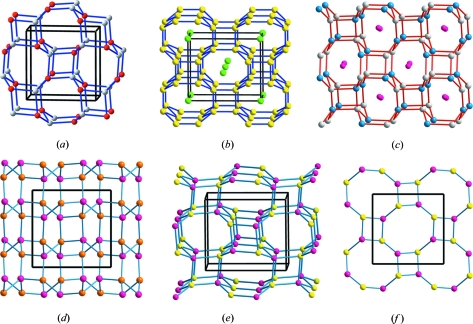
The structures formed by four-connected networks, involving (II)–(VI), (III)–(V) and (IV)–(IV) pairs of atoms. (*a*) The structure of β-BeO. (*b*) Perspective view of the tetrahedral B network in CrB_4_ (Cr atoms as green spheres). (*c*) The AlSi network in anorthite (CaAl_2_Si_2_O_8_). Ca atoms are lodged in the octagonal tunnels. (*d*) The AlP subarray of variscite AlPO_4_·2H_2_O. Note the similarity with the HP-Si of Fig. 1 ▶(*b*). The Al and P atoms form the puckered **4.8^2^** layers. (*e*) The AlP subarray in metavariscite. Note the differences in the stacking of the **4.8^2^** layers in variscite and, at the same time, the similarities with both anorthite and β-BeO. (*f*) An isolated puckered **4.8^2^** layer to show its differences with the networks of HP-Si represented in Fig. 1 ▶(*e*).

The structure is also related to the four-connected networks of β-BeO (Smith *et al.*, 1965 ▶), CrB_4_ (Andersson & Lundström, 1968 ▶), anorthite (CaAl_2_Si_2_O_8_; Takeuchi *et al.*, 1973 ▶) and the AlP sub-arrays of two polymorphs of AlPO_4_·2H_2_O (the minerals variscite and metavariscite; Kniep & Mootz, 1973 ▶; Kniep *et al.*, 1977 ▶; Kniep, 1978 ▶). In all these structures, represented in Fig. 2 ▶, the involved atoms form puckered layers of octagons and squares (the **4.8^2^** nets), instead of the helices existing in the γ-Si(Ge) structure. Compare the structures drawn in Figs. 1 ▶ and 2 ▶.

#### 2.1.1. Li_3_AlN_2_

The structure of Li_3_AlN_2_ was reported exactly 60 years ago by Juza & Hund (1948 ▶). It can be analysed in the light of both the Zintl (1934 ▶) and Klemm (1958 ▶) concepts, in terms of which, various electron redistributions are allowed, provided that the overall electron count remains the same. Table 2 ▶ summarizes some of the possible redistributions for those species relevant to the present work.

*Case 1*: For example, if the formula Li_3_AlN_2_ were to be expressed in the usual ionic form, it would be written as (Li^1+^)_3_[AlN_2_]^3−^, *i.e.* 3 Li^1+^ cations and the [AlN_2_]^3−^ anion. If, then, two Li atoms were to donate two valence electrons to the two N atoms, converting them into two (Ψ-O) atoms, and the third Li atom transferred its electron to the Al atom, converting it into (Ψ-Si), the formal outcome would be (Li^+1^)_3_[Al^−1^(N^−1^)_2_] or (Ψ-He)_3_(Ψ-Si)(Ψ-O)_2_ (Table 2 ▶): this pseudo-compound would then be expected to adopt the tetrahedral structure of one of the phases of elemental Si, which would explain why the Al atom (Ψ-Si) occupies the 16*c* site of the structure.

**Figure 3:**
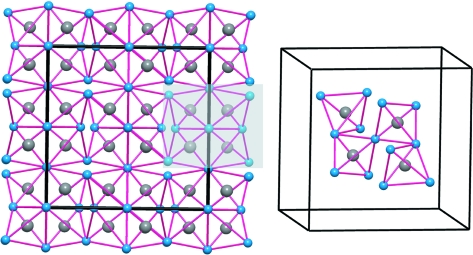
(*a*) The AlN_2_ substructure of the stuffed bixbyite Li_3_AlN_2_ formed by a three-dimensional network of AlN_4_ tetrahedra which share all corners. (*b*) Detail of the four central tetrahedra to show their connectivity.

Effectively, the Al^−1^ (Ψ-Si) atoms form the expected four-connected skeleton and the [AlN_2_]^−3^ substructure becomes a (Ψ-SiO_2_)-like network of AlN_4_ tetrahedra, sharing all four corners, as found in most structural variants of silica. The result is that this may be regarded as Al-stuffed anti-bixbyite (Li_3_N_2_), with the substructure shown in Fig. 3 ▶ (Niewa *et al.*, 2003 ▶). This feature is also in agreement with the ‘general principle’ deduced in the recent work of Vegas & García-Baonza (2007 ▶), by which atoms try to form pseudo-structures characteristic of their group 14 isoelectronic counterparts.

**Figure 4:**
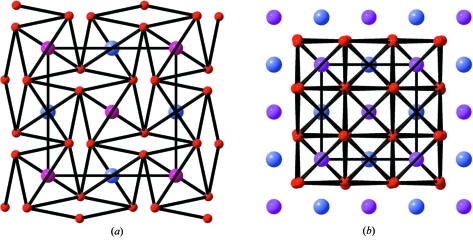
(*a*) The structure of BPO_4_ (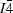) at 6.1 GPa, projected on the *ab* plane. It is cristobalite-like and, under pressure, undergoes a continuous tilting of the B(P)O_4_ tetrahedra up to collapse, at 50 GPa, into an almost regular f.c.c. array of the O atoms represented in (*b*). This final step corresponds to the chalcopyrite structure of CuFeS_2_. Note that the rhombs seen in (*a*) are equivalent to those described in Fig. 2 ▶(*a*). In this continuous tilting of tetrahedra, the cation array BP remains as a blende-type structure.

*Case 2*: The structure can accommodate a second interpretation (Table 2 ▶). If the Zintl–Klemm concept were applied in the opposite direction, the Al atom would transfer its three valence electrons to Li forming three Li^−1^ species which then become isoelectronic with three (Ψ-Be): this would leave an [AlN_2_]^+3^ component made up of Al^+3^ and (N^0^)_2_. Li^−1^ is (Ψ-Be), Al^+3^ is (Ψ-Ne), and N^0^ remains as N. The result is an Al^+3^-stuffed Li_3_N_2_ or a hypothetical (Ψ-Ne)-stuffed (Ψ-Be_3_N_2_) with the anti-bixbyite structure, like the real Be_3_N_2_ compound (von Stackelberg & Paulus, 1933 ▶). This description of bixbyite is illustrated in Fig. 3 ▶(*a*), where the N atoms (blue spheres) are arranged in an almost f.c.c. array. To see the deviation from the ideal f.c.c. array, we have represented in Fig. 4 ▶(*a*) a similar array of O atoms observed in BPO_4_ (Haines *et al.*, 2003 ▶). Although the structures are not identical, they can be compared on the basis of the similar O sub-array, and also because both compounds are formed by *X*O_4_ tetrahedra. In this compound (cristobalite-like), the P(B)O_4_ tetrahedra rotate in a continuous way when pressure is applied. At 50 GPa, the O array collapses, as in the chalcopyrite structure (FeCuS_2_), into an almost perfect f.c.c. arrangement, represented in Fig. 4 ▶(*b*) (Haines *et al.*, 2003 ▶).

*Case 3*: Yet a third interpretation is possible (Table 2 ▶). This arises from the fact that the overall structure is essentially anti-fluorite, N_2_ *M*_4_, with N*M*_8/4_ cubes sharing edges, where *M*_4_ = Li_3_Al. A well known example is Mg_2_Si in which SiMg_8/4_ cubes share edges (Owen & Preston, 1924 ▶).

If we assume the transfer of two electrons from the two N atoms to the [Li_3_Al] array, the two N atoms each become N^+1^ or (Ψ-C), while the [Li_3_Al]^−2^ array is electronically equivalent to (Ψ-Li_3_P) with its total of eight valence electrons for the four cations. This pseudo-compound (Ψ-Li_3_P) is then electronically equivalent to either (Ψ-Mg_4_) or (Ψ-Be_4_). Consequently, the compound Li_3_AlN_2_ would be formulated as the hypothetical (Ψ-Li_3_PC_2_), electronically equivalent to (Ψ-Mg_4_Si_2_) – the real anti-fluorite is Mg_2_Si (Owen & Preston, 1924 ▶). However, this structure is no longer a stuffed anti-bixbyite. On the other hand, the hypothetical (Ψ-Li_3_PC_2_) compound could be described as a hypothetical, phosphorus-stuffed Li_3_C_2_ with the anti-bixbyite type structure.

The Zintl–Klemm concept can account for even more pseudo-structures derived from Li_3_AlN_2_.

**Figure 5:**
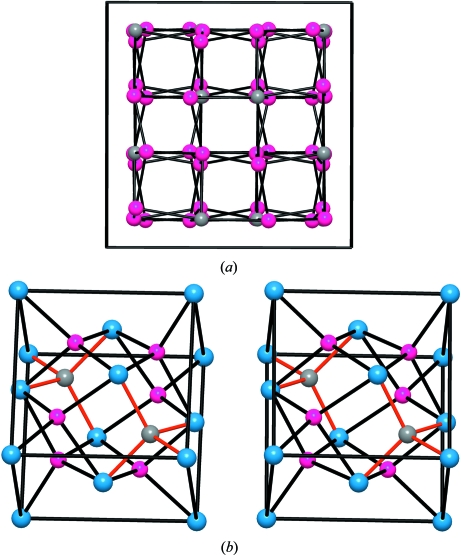
(*a*) The Li_3_Al(Ge) subarray projected on the *ab* plane. They form a distorted simple cubic array. As in CaF_2_, the N atoms (omitted) occupy alternate cubes of this AlLi_3_ array. The N atoms form, in turn, a distorted f.c.c. structure like In atoms in In_2_O_3_. (*b*) Stereopair representing a distorted face-centered cube of N atoms (blue spheres) with all the tetrahedral holes occupied by Li (pink) and Al(Ge) (grey) atoms. The structure is a distorted fluorite-like array where the ZnS-blende structure is also implicit. Note the special location of Ge(Al) atoms.

**Figure 6:**
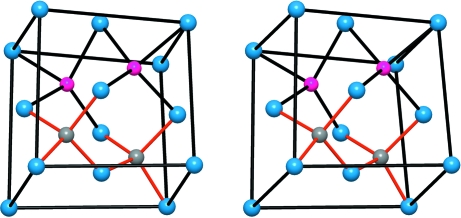
A fragment of the structure of Li_3_AlN_2_. The stereopair shows a distorted f.c.c. unit cell of N atoms (blue spheres) with half of the tetrahedral holes occupied by two Al atoms (grey spheres) and two Li atoms (Ψ-B) (pink spheres). All together they form a unit cell of the blende-type AlBN_2_ (a III–V compound). The remaining four Li atoms, considered as donors, have been omitted. The distorted f.c.c. array of N atoms and the unit cell of elemental Ne have similar dimensions.

*Case 4*: By assuming that two Li atoms donate 2*e* ^−^ to the third Li atom to give (Li^+1^)_2_[Li^−2^AlN_2_]^−2^, Li^−2^ would convert into (Ψ-B), the anion becoming Ψ-[BAlN_2_]. We must recall that both AlN and BN (III–V compounds) form blende-type structures (Wakatsuki *et al.*, 1972 ▶) that are implicit in Li_3_AlN_2_, *i.e.* in its constituent nitrides Li_3_N and AlN (see Fig. 5 ▶ *b*). When all the Li atoms (donors and acceptors) are drawn (Fig. 4 ▶ *b*), the generated structure is a unit cell of a distorted fluorite, as was shown in Fig. 5 ▶. If four of the six Li atoms (the donors) were eliminated, the blende-type pseudo-array, of the formula (Ψ-LiAlN_2_), would become evident, as seen in Fig. 6 ▶.

A final comment should be made on the N array. It has been shown (Figs. 3 ▶ and 5 ▶) that N atoms form a slightly distorted f.c.c. array, as do the indium atoms in In_2_O_3_ (Ramos-Gallardo & Vegas, 1995 ▶). If we assume that all 6 electrons from [Li_3_Al] are transferred from these cations to form two nitride N^3−^ anions, the latter would behave as (Ψ-Ne). The distorted f.c.c. unit cells of N atoms have dimensions varying from 4.82 to 4.89 Å (mean 4.85 Å). This value is close to that of the unit cell of elemental Ne, also f.c.c., with *a* ≃ 4.50 Å. The N—N distances, in the partial LiAlN_2_ array, range from 3.11 to 3.14 Å, close to the Ne—Ne distances (3.18 Å) in elemental Ne. Thus, the Ne structure can also be recognized in Li_3_AlN_2_ (see Fig. 5 ▶).

This last compound provides a nice example of how the location of atoms in different structural sites is not only determined by their relative atomic sizes, but also by their pseudo-electronic configurations. It could be said that such compounds highlight the unequivocal relationship existing between composition and structure which was postulated by Vegas & García-Baonza (2007 ▶).

#### 2.1.2. Li_3_[(*M* ^IV^)_0.67_Li_0.33_]N_2_

*Case 5*: The compounds, Li_3_[(*M* ^IV^)_0.67_Li_0.33_]N_2_, follow a similar pattern. Here, the 16*c* site of space group 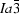 is randomly occupied by *M* ^IV^ (Ge) and Li atoms, with reported population parameters of 0.67 and 0.33 for *M* ^IV^ and Li, respectively. The reader can readily recognize that this composition is equivalent to the presence of an atom with three valence electrons, like Al in Li_3_AlN_2_ (Juza & Hund, 1948 ▶). Thus, the fractional occupation factors are explained by the need for satisfying the four valence electrons of the 2/3 *M* ^IV^ plus 1/3 Li atoms located at 16*c*. Thus, two of the three Li atoms located at 48*e* transfer two electrons to the N atoms, converting them into two (Ψ-O). The third Li atom at 48*e* (16 Li atoms) donates its valence electron to the Li_0.33_ located at 16*c* to generate (Li^+1^)_3_(Li^−3^)_0.33_(*M* ^IV^)
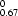
(N^−1^)_2_, with the electronically balanced charge distribution of Li_3_AlN_2_. This transfer of three electrons to the core Li atom converts it into (Ψ-C), hence forming, together with the *M* ^IV^ atoms, a four-connected net. Now we are able to explain two structural features: on the one hand, why the Li atoms occupy one third of the 16*c* site and not another position such as 8*b*. The reason is that this is the only way of achieving the observed four-connected network of γ-Ge. We can also explain why the *M* ^IV^ atoms are precisely located at that site and not partially occupying the alternative 48*e* positions: in this latter site, the Ge skeleton could not be formed.

#### 2.1.3. Li_6_[*M* ^V^Li]N_4_ (*M* ^V^ = V, Nb, Ta)

**Figure 7:**
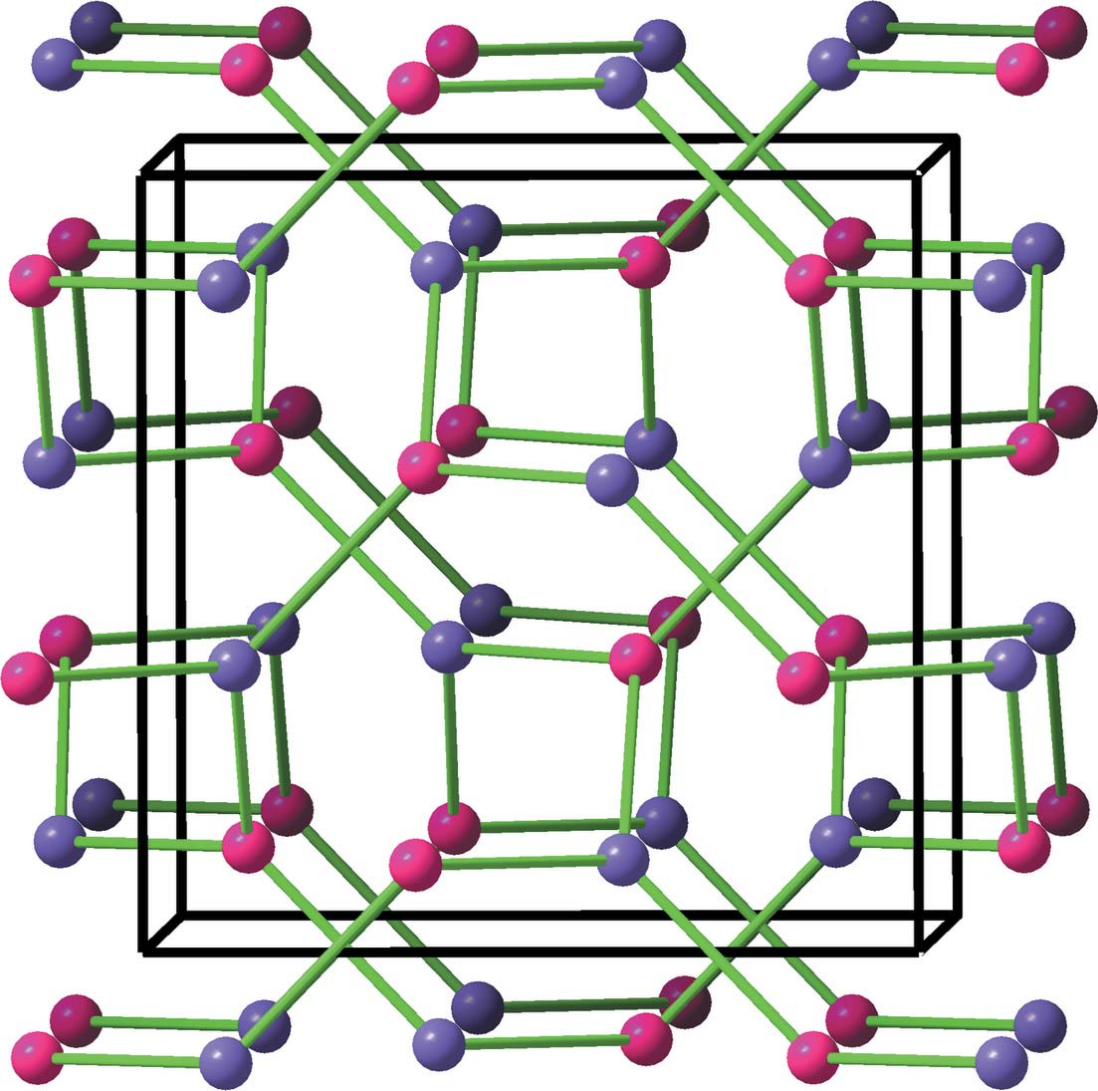
The [VLi] skeleton in Li_6_[LiV]N_4_ similar to both the Al(Ψ-Si)-array in Li_3_AlN_2_ and the structure of γ-Si, represented in Fig. 1 ▶. Li and V atoms are ordered occupying different positions of the 8*c* site in the space group 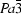. Each atom connects to four unlike atoms. One of these bonds has been omitted to show the two interpenetrating three-connected subsets.

*Case 6*: Like Li_3_AlN_2_ and Li_3_[Li_0.33_Ge_0.67_]N_2_, the EZKC, when applied to this compound, predicts that it also involves four-connected nets. For example, at one analytical level we can suppose that four Li atoms donate four electrons to the N atoms, converting them into (Ψ-O), and that the two remaining Li atoms transfer two additional electrons to the [*M* ^V^Li] set, converting it into either (Ψ-[III–V]) or (Ψ-[II–VI]) pairs, forming four-connected nets. The [*M* ^V^Li]^−2^ substructure is represented in Fig. 7 ▶. Here, the *M* ^V^ and Li atoms are not distributed at random, as in the 16*c* site of 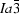, but they are ordered into two sets on 8*c* sites of the subgroup 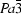 from which the body-centering operation in 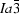 has been dropped.

At a slightly deeper level, the outcome of these electron transfers (see Table 2 ▶) is the compound (Li^+1^)_3_(Li^−3^)_0.5_(*M* ^+1^)_0.5_(N^−1^)_2_, which is isostructural with the four-connected network of the tetrahedral pseudo-compound (Ψ-He)_3_(Ψ-C_0.5_Ti_0.5_)(Ψ-O)_2_ (assuming that *M* ^+1^ = V^+1^), *i.e.* the hypothetical (Ψ-He_3_[C_0.5_Ti_0.5_]O_2_), a hypothetical ‘C/Ti-stuffed He_3_O_2_’. Again, we have what is essentially a V/Li-stuffed Li_3_N_2_.

The discussion about the different structures which co-exist in these stuffed bixbyite-type compounds can be extended to these compounds. The lack of isostructural compounds containing Group 15 elements (P, As, Sb and Bi) is noteworthy. However, we must recall the similar chemical and structural behaviors of vanadates and niobates on one hand, and phosphates and arsenates on the other. Moreover, if we assume that the [*M* ^V^Li]^−2^ sub-array is formed by a (Ψ-[III–V]) pair, it must be strongly related to [AlP] substructures like those of AlPO_4_, represented in Fig. 2 ▶. Compare Fig. 2 ▶(*d*) of variscite with Fig. 7 ▶. The remaining co-existing structures can be easily deduced, as with Li_3_AlN_2_.

Table 3 ▶ attempts to collect and summarize all the foregoing discussion of the Zintl–Klemm analyses.

### 2.2. Coordination-defect (CD) approach

The CD theory was proposed originally by Martin (1974 ▶) to describe the structures of the many well known, anion-deficient, fluorite-related compounds, of which bixbyite is but one example. He proposed that the anion vacancies in such structures were not simply isolated point defects, randomly distributed throughout the fluorite lattice, but rather that each vacancy was strongly coordinated by its nearest and next-nearest neighbours, four cations and six anions, to generate an octahedral structural entity, the CD, of considerable thermodynamic and structural stability. Indeed this concept can be extended to other non-fluorite structures of different symmetry for it can be argued that the same principle would hold true for the presence of any atom different in kind from the predominant atomic species forming a lattice.

**Figure 8:**
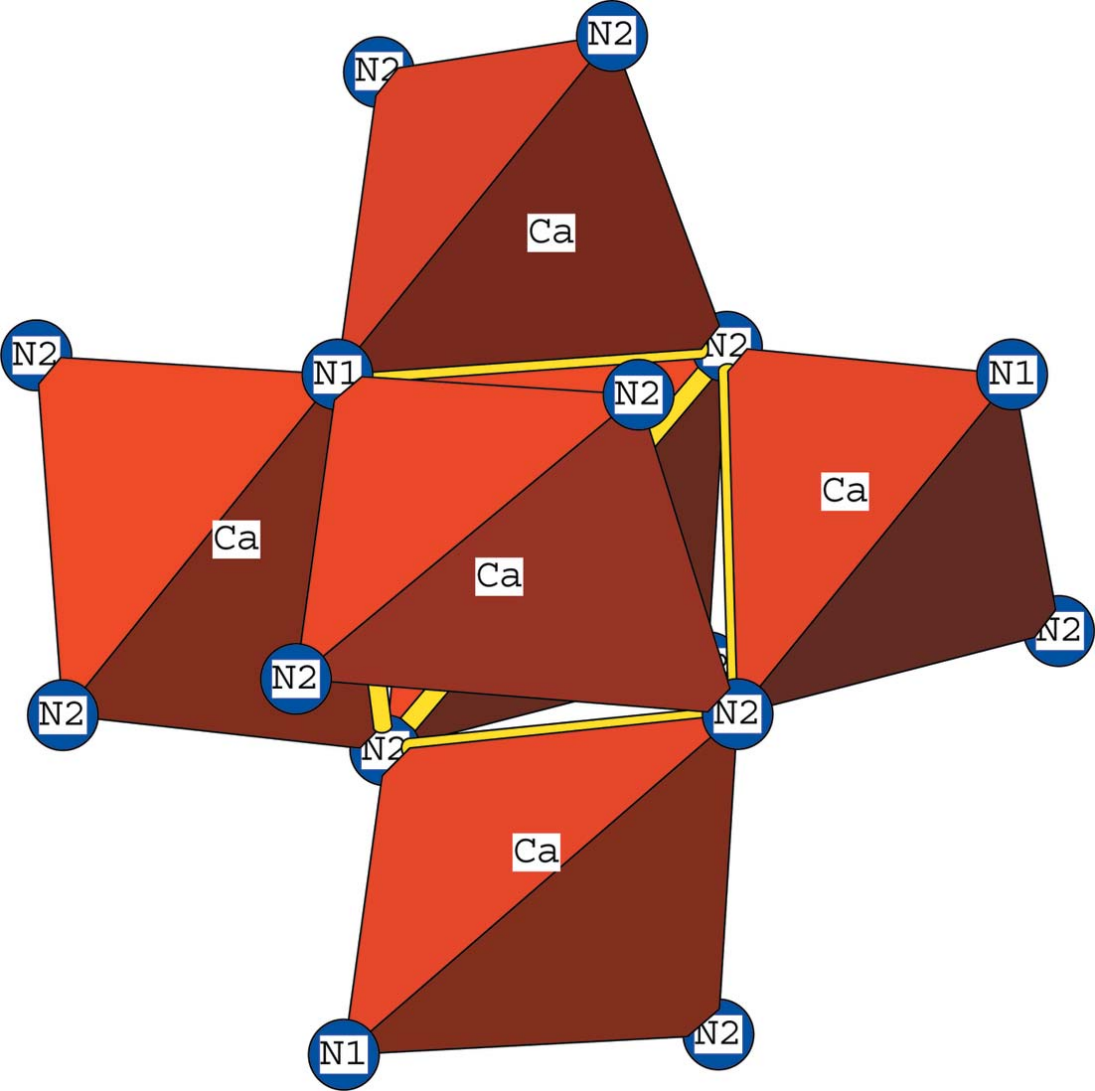
The anti-CD in Ca_3_N_2_. The □_*M*_ *X*_4_ tetrahedron at the centre is drawn in yellow outlines. The six peripheral CaN_4_ tetrahedra are drawn as such in red.

In the context of an anti-bixbyite structure (space group 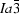) such as magnesium nitride (a cation-deficient, anti-fluorite-related compound of the general formula *M*_3_□_*M*_N_2_, where □_*M*_ is a vacancy in the cation sublattice), what we now might call an anti-CD is described as an octahedron of *MX*_4_ tetrahedra sharing corners to enclose an empty tetrahedron (□*_M_X*_4_) or, alternatively, an empty tetrahedron, (□*_M_X*_4_), sharing all six edges with *MX*_4_ tetrahedra, forming a □_*M*_-centred octahedron represented as *M*_6_□_*M*_ *X*_4_. This is shown in Fig. 8 ▶, comprising a central tetrahedral core with six peripheral tetrahedra.

Incorporated into the anti-fluorite-type structure, and therefore taking account of the sharing of all tetrahedral edges such that every *X* atom is common to eight tetrahedra, the anti-CD composition becomes □*_M_X*_4/8_(*MX*_4/8_)_6_ or *X*_7_ *M*_12_(□_*M*_)_2_. Moreover, analogous to the bixbyite case, each peripheral *MX*_4/8_ tetrahedron of the anti-CD in the anti-bixbyite structure is common to another anti-CD, and the anti-bixbyite composition expressed in the anti-CD format is □*_M_X*_4/8_(*MX*_4/8_)_6/2_ or *M*_3_□_*M*_ *X*_2_. The □_*M*_ site is the 16*c* site of 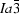, which is also the site occupied by Si in γ-Si. If, now, this vacancy can accommodate a pseudo-Si atom, we obtain the compound *M*_3_(Ψ-Si)*X*_2_, whose counterparts are Li_3_AlN_2_ and Li_3_ScN_2_, with a truly ‘stuffed’ anti-bixbyite structure, the Al and Sc atoms occupying respectively the tetrahedrally coordinated, vacant cation sites of the actual anti-bixbyite structure.

In the case of Li_3_AlN_2_, and assuming the normal ionic charge assignments, the anti-CD core becomes the [Al^3+^(N^3−^)_4/8_]^1.5+^ tetrahedron, with Al occupying the anti-bixbyite cation vacancy, while each of the six peripheral anti-CD tetrahedra in the overall structure is [(Li^1+^)(N^3−^)_4/8_]^0.5−^. In standard chemical usage, the overall anti-CD formula of Li_3_AlN_2_ is (AlN_4/8_)^1.5+^[(LiN_4/8_)]_6/2_]^1.5−^.

*Case 1*: We now apply the first of the above Zintl–Klemm interpretations of Li_3_AlN_2_, *i.e.* two Li atoms donate two valence electrons to the two N atoms converting them into (Ψ-O) atoms, the third Li atom transfers its electron to the Al atom, converting it into (Ψ-Si). The formal outcome is (Li^+1^)_3_Al^−1^(N^−1^)_2_ in the Zintl–Klemm notation. Here, Li^+1^ is (Ψ-He), Al^−1^ and N^−1^ are (Ψ-Si) and (Ψ-O), respectively. The anti-CD formulation of this becomes [Al^−1^(N^−1^)_4/8_]^−1.5^ as the core, the peripheral tetrahedra being [Li^+1^(N^−1^)_4/8_]^+0.5^, with Al^−1^, *i.e.* (Ψ-Si), ‘stuffing’ the tetrahedral void, □_*M*_, in the anti-CD core. Each of the six peripheral tetrahedra of the anti-CD, with the formula [Li^+1^(N^−1^)_4/8_]^+0.5^, is common to another such anti-CD, and has Li^+1^, with the (Ψ-He) spherical electron configuration at its centre, coordinated by a tetrahedron of N^−1^ atoms, *i.e.* (Ψ-O). The resulting pseudo-anti-CD representation of Li_3_AlN_2_ can now be written as [Al^−1^(N^−1^)_4/8_]^−1.5^{[Li^+1^(N^−1^)_4/8_]^+0.5^}_6/2_ or [(Ψ-Si)(Ψ-O)_4/8_][(Ψ-He)(Ψ-O)_4/8_)]_6/2_, *i.e.* overall (Ψ-SiO_2_) + 3(Ψ-He) with the spherical eight-electron configuration. This is equivalent to (Ψ-He_3_SiO_2_), *i.e.* a hypothetical (Ψ-Si)-stuffed anti-bixbyite, in agreement with the conclusion reached in the previous section. In this CD description, we see that the CD core has an excess of electrons, while the core periphery has an equal electron deficit.

*Case 2*: The second interpretation applies the Zintl–Klemm concept in the opposite direction, with the Al atom transferring its three valence electrons to Li forming three Li^−1^ species, each of which is isoelectronic with (Ψ-Be) and an [Al^+3^(N^0^)_2_]^+3^ sub-structure yielding a (Ψ-Ne)-stuffed (Ψ-Be_3_N_2_), with the anti-bixbyite structure as in real Be_3_N_2_ (von Stackelberg & Paulus,1933 ▶). With this second set of electronic assignments, Li_3_AlN_2_ can be formulated as the anti-CD, [Al^+3^(N^0^)_4/8_]^+3^{[Li^−1^(N^0^)_4/8_]^−1^}_6/2_. The pseudo-anti-CD can now be written as [(Ψ-Ne)N_4/8_][(Ψ-Be)N_4/8_]_6/2_, *i.e.* overall (Ψ-Be_3_NeN_2_) – a hypothetical, neon-stuffed (Ψ-Be_3_N_2_), as described earlier.

*Case 3*: The third interpretation involves the transfer of two electrons from the two N atoms to the Al so that the subarray becomes [Al^−2^(N^+1^)_2_]^0^, which is electronically equivalent to (Ψ-P)(Ψ-C)_2_. The CD formulation becomes [Al^−2^(N^+1^)_4/8_]^−1.5^{[Li^0^(N^+1^)_4/8_]_6/2_}^+1.5^, with the corresponding pseudo-CD being [(Ψ-P)(Ψ-C)_4/8_][Li^0^(Ψ-C)_4/8_]_6/2_ or (Ψ-Li_3_PC_2_), *i.e.* a phospho-carbon stoichiometric analogue of Li_3_AlN_2_. Indeed it can be described as a hypothetical phosphorus-stuffed lithium carbide, Li_3_PC_2_ with the anti-bixbyite structure. These descriptions are summarized in Table 4 ▶.

*Case 4*: This case, discussed above, identifies the tetrahedral blende-type structures, AlN and BN, that are implicit in Li_3_AlN_2_. Thus, the electron distribution (Li^+1^)_2_[Li^−2^AlN_2_], as quoted above, leads to the pseudo compound, (Ψ-He)_2_[(Ψ-B)AlN_2_], confirming a blende-type anion which could be regarded as a 1:1 mixture of (Ψ-He)(Ψ-BN) and (Ψ-He)(Ψ-AlN).

Since the stoichiometry of blende-type structures is quite different from that of anti-bixbyite, the CD concept is no longer relevant. However, it is encouraging that the CD formulations of the first three interpretations are completely consistent with those derived by employing the Zintl–Klemm concept.

*Case 5*: Li_3_[(*M* ^IV^)_0.67_Li_0.33_]N_2_. Apart from the disordered mix of *M* ^IV^ and Li within the core tetrahedron, the treatment of this phase is the same as for Li_3_AlN_2_. This is summarized in Table 5 ▶.

*Case 6*: In the case of the Li_6_[*M* ^V^Li]N_4_ compounds, because the set in the square brackets is strictly ordered, there will be two kinds of anti-CD tetrahedral core, [LiN_4/8_] and [*M* ^V^N_4/8_], and just the one type of peripheral tetrahedron, [LiN_4/8_]. The composition of the compound Li_6_[Li*M* ^V^]N_4_ can also be written as Li_3_[Li_0.5_ *M* 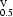]N_2_. This ensures that the fractional population parameters associated with Li^I^ and *M* ^V^ (= V, Nb, Ta) continue to provide an electronically balanced charge distribution. As with Li_3_AlN_2_ and Li_3_[Li_0.33_Ge_0.67_]N_2_, the Zintl–Klemm concept, when applied to this compound, confirms that it also involves 4-connected nets. For example, if three electrons were transferred from Li_3_ to the two N and the single core-Li atom, the species (Li^+1^)_3_, Li^−1^ in the core, and (N^−1^)_2_ would result. If an additional electron were transferred from *M* ^V^ to the core Li atom, the resulting compound would become (Li^+1^)_3_[(Li^−3^)_0.5_(*M* ^+1^)_0.5_](N^−1^)_2_, which is isostructural with the tetrahedral pseudo-compound (Ψ-He)_3_[(Ψ-C)_0.5_(Ψ-*M* ^IV^)_0.5_](Ψ-O)_2_ and its four-connected tetrahedral network. If we rewrite this as (Li^+1^)_6_[Li^−3^ *M* ^+1^](N^−1^)_4_, this would lead to two types of anti-CD, one being [(Li^−3^)(N^−1^)_4/8_]^3.5−^{[(Li^+1^)(N^−1^)_4/8_]^+0.5^}_6/2_, with a total charge of −2, *i.e.* [(Ψ-C)(Ψ-O)_4/8_](Ψ-He)(Ψ-O)_4/8_, and the other [*M* ^+1^(N^−1^)_4/8_]^+0.5^{[(Li^+1^)(N^−1^)_4/8_]^0.5+^}_6/2_, with a total charge of +2.0, *i.e.* (Ψ-*M* ^IV^)(Ψ-O)_4/8_(Ψ-He)(Ψ-O)_4/8_. Thus, there is overall charge balance. This result is consistent with our earlier comment that the Li and *M* ^V^ atoms constituting the CD cores are highly ordered, thereby ensuring that the positive and negatively charged anti-CDs are appropriately disposed with respect to their opposed anionic and cationic charges. See Table 5 ▶.

## 3. Concluding remarks

### 3.1. Need for EZKC approach

In this paper we have explored a new application of the Zintl–Klemm concept. It is well known that the classical Zintl–Klemm concept (ZKC) was enunciated to account for the so-called Zintl phases in which electron transfer, from very electropositive cations to atoms of the *p*-block elements, leads to the formation of Zintl polyanions. The structure of the compound NaSi, based on tetrahedral Si_4_ groups, illustrates very clearly how the Si atom is converted into Si^−1^ (Ψ-P) to form (Ψ-P)_4_ molecules. Recently, Santamaría-Pérez & Vegas (2003 ▶) and Vegas & García-Baonza (2007 ▶) have shown that the Zintl–Klemm concept can be extended to the cation arrays of inorganic compounds. This extension of the ZKC (EZKC) necessitates charge transfer between cations, even if they are of the same atomic species. This unusual extension then ensures that this wider application of the general principle continues to be valid. The principle states that, in many compounds, the electron configurations of pairs of atoms can be rearranged to generate the characteristic structures of the Group 14 elements (Vegas & García-Baonza, 2007 ▶).

The novelty of the present contribution is that in the case of the anti-bixbyite-type compounds derived from lithium nitride, the nitrogen can also play a central role in the interpretation and rationalization of these structures. Thus, traditional anions and cations together can be involved in the charge-transfer process in order to produce a variety of possible structures. With the compounds discussed here it is clear that the application of this extended principle can now explain why several structure types – fluorite, the hypothetical blende AlBN_2_ (*i.e.* the real blendes AlN and BN), the real anti-bixbyite Mg_3_N_2_ (von Stackelberg & Paulus, 1933 ▶) and the real γ-Si (Kasper & Richards, 1964 ▶) – are all identifiable in Li_3_AlN_2_ and its isotypes. Thus, a novel conclusion of this study is that all these structure types are satisfied at the same time, the Zintl–Klemm concept being the universal key. In other words, a given compound might result from multiple resonance structures, which implies a partial delocalization of electrons. When these are distributed over all the atoms, the electron-count requirements for each structure are fulfilled. This would be a new convergence point between solid state and molecular chemistry.

In the case of the quaternary compounds Li_3_(Ge_0.67_Li_0.33_)N_2_ and Li_3_(Ti_0.67_Li_0.33_)N_2_ (Juza *et al.*, 1953 ▶), we have provided arguments to demonstrate that both the relative amounts and the exact positioning of the Ge(Ti) and Li atoms inside the brackets are not just coincidental.

**Figure 9:**
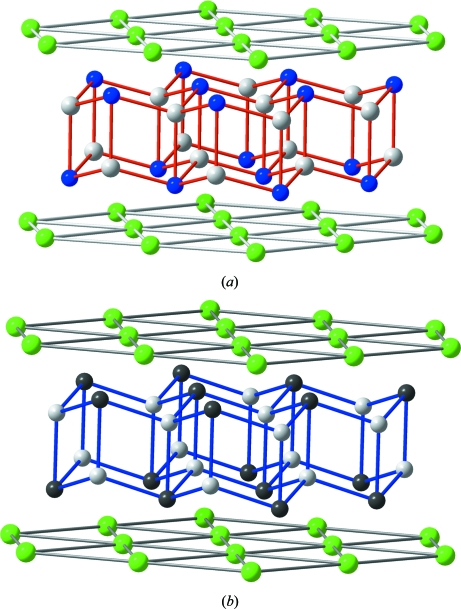
(*a*) The structure of CaMg_2_N_2_. The hexagonal close packed (h.c.p.) layers of the Ca atoms (green spheres) are perpendicular to **c**. Both Mg and N atoms form bilayers in which all atoms are four-connected. The Mg atoms (grey spheres) are at the center of the regular tetrahedra. The N atoms (blue spheres) connect to four Mg atoms forming an umbrella-like, inverted tetrahedron. (*b*) The equivalent structure of CaAl_2_Si_2_. Grey and black spheres represent Al and Si atoms, respectively.

The correlation of Li_3_AlN_2_ with the tetrahedral blende structures necessitates an unusual and unsymmetrical charge transfer, [Li^0^]_3_ → [(Li^+1^)_2_Li^−2^], with two Li atoms donating one electron each to the third Li atom to give (Li^+1^)_2_[Li^−2^AlN_2_]. Li^+1^ is (Ψ-He); Li^−2^ is (Ψ-B), so that the anion becomes (Ψ-BAlN_2_), providing the rationale for the presence of the blende-type anion in the pseudo compound, (Ψ-He)(Ψ-BAlN_2_) represented in Fig. 6 ▶. This could be viewed as a controversial proposal in the case of an electropositive element such as lithium: however, such unsymmetrical charge transfers are not particularly uncommon (Vegas & García-Baonza, 2007 ▶). For example, both Ca_3_N_2_ and Mg_3_N_2_ (von Stackelberg & Paulus, 1933 ▶) possess anti-bixbyite structures, and, in line with the arguments developed above, the identification of a blende structure in Mg_3_N_2_, for example, can be explained by assuming that one Mg atom transfers its two electrons to the other two Mg atoms so that Mg_3_ in Mg_3_N_2_ becomes Mg^+2^(Mg^−1^)_2_. Mg^+2^ is (Ψ-Ne), Mg^−1^ is (Ψ-Al), leading to the overall composition (Ψ-Ne)(Ψ-Al)_2_N_2_. The same description can be applied to Ca_3_N_2_, although the similar ternary, mixed-cation compounds, such as CaMg_2_N_2_ (Schulz-Coulon & Schnick, 1995 ▶) and BaMg_2_P_2_ (Klüfers & Mewis, 1984 ▶), no longer conform to this intra-cation transfer. Thinking in classical terms of structures dominated by ionic charge and size effects, and conventional coordination polyhedra, it might be expected that the three *M* cations [CaMg_2_, BaMg_2_] would either randomly occupy the 48*e* site of 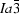 or be ordered into a superstructure. However, the structure is in fact no longer anti-bixbyite, but one related to that of the Zintl phase Ca[Al_2_Si_2_] (Gladyshevskii *et al.*, 1967 ▶), which is represented in Fig. 9 ▶.

The solution found by nature is quite elegant: because both Ca and Ba are more electropositive than Mg, they donate their two valence electrons to the two Mg atoms, converting each into (Ψ-Al), which, together with the N(P) atoms, form a four-connected (Ψ-AlN) (Ψ-AlP) network typical of the [Al_2_Si_2_]^2−^ Zintl polyanion, and hence of the Group 14 elements. The same solution was found for the compound ZrNCl (Vegas & Santamaría-Pérez, 2003 ▶) in which the [ZrN] array transfers one electron to the Cl atom, giving rise to the polycation [ZrN]^+1^, isoelectronic with Ψ-YN. This is a good example of how the electron transfer acts (in this case, in the opposite direction), to produce ‘Zintl polycations’.

### 3.2. Experimental justification

It is important to recall that such unexpected electron transfers are supported by NMR experiments that indicate the co-existence of entities such as K^+^ and K^−^ (*e.g.* potassides), even in the solid state (Tinkham & Dye, 1985 ▶; Nakayama *et al.*, 1994 ▶; Terskikh *et al.*, 2001 ▶), so it is quite conceivable that these ions might coexist in other compounds, regardless of the fact that such entities could not be identified in conventional diffraction experiments (Seiler & Dunitz, 1986 ▶).

In our opinion, the most remarkable finding of Niewa *et al.* (2003 ▶) was the coincidence of the γ-Si(Ge) structure with the Sc sub-array, and the consequent existence of the tetrahedral ScN_2_ skeleton, with covalent Sc—N bonds. Theoretical calculations, based on the electron localization function (ELF) and periodic nodal surfaces (PNS), indicated that Li_3_ScN_2_ is formed by Li^1+^ cations inserted in a three-dimensional skeleton of [ScN_2_]^3−^. The authors also mention the isostructural nitrides Li_3_AlN_2_ and Li_3_GaN_2_. It is noteworthy that this insight is only one of the many we have discovered in these stuffed bixbyites. From this point of view, the fact that the compound forms this type of structure remains unexplained. The coexistence of Li^1+^ cations and the tetrahedral polyanion [ScN_2_]^3−^ could have been achieved with any of the many silica-like skeletons.

We have already mentioned that the isostructural compound Li_3_(Ge_0.66_Li_0.33_)N_2_ (Juza *et al.*, 1953 ▶) is the key to understanding this family of compounds. However, this compound was overlooked by the authors (Niewa *et al.*, 2003 ▶). As explained above, this compound provokes two crucial questions:

(i) Is it possible to explain a γ-Ge-type substructure for the (Ge_0.66_Li_0.33_) set on the basis of the arguments given by Niewa *et al.* (2003 ▶)?
(ii) Why are the Ge atoms at the 16*c* positions and not distributed at random over the other possible Li positions as in (Li_2.33_Ge_0.66_)LiN_2_?

In fact, in this latter case the anti-fluorite-type structure remains and the coordination number for all the atoms is the same. Our investigation began because we expected that a Ge(Si) atom must be at this 16*c* position, reproducing the structure of elemental Ge, and indeed the Ge atoms are there.

### 3.3. New perspectives

Inorganic solids should actually be regarded from a holistic perspective. One of the few models that can provide this holistic view is the Zintl–Klemm concept (ZKC) and its extension the EZKC. Until recently, this long-standing and illuminating concept has only been applied to the so-called Zintl phases. However, we have shown that this approach also applies successfully to the cation arrays in aluminates and silicates (Santamaría-Pérez & Vegas, 2003 ▶; Santamaría-Pérez *et al.*, 2005 ▶), putting this multitude (thousands of compounds), for the first time, on a common and rational basis. In a more recent article, it has been shown that the ZKC can go even further (Vegas & García-Baonza, 2007 ▶) and provide a rational explanation of the many and varied structural types. Within this emerging pattern is where the novelty of this article resides. Here we provide a chemical reason for the experimental observation that the Sc sub-array in Li_3_ScN_2_ resembles the tetrahedral structure of γ-Si(Ge). There is a huge conceptual difference between simply pointing out this topological similarity, and describing the structure by using the ZKC. The latter enables us to explore the structures of all the likely contributing resonance compounds discussed above. For example, we are able to give a rational explanation as to why the Sc atoms are tetrahedrally coordinated in Li_3_ScN_2_, whereas they are octahedrally coordinated in the so-called interstitial nitride ScN? ZKC analysis leads us to [Sc^+2^N^−2^], *i.e.* the pseudo-compound (Ψ-KF) with the NaCl cubic close-packed structure.

In Li_3_ScN_2_, theoretical calculations, especially the periodic nodal surfaces, have led to the interpretation of the structure as composed of Li^+^ cations and (ScN_2_)^3−^ polyanions. However, the question is how to account for the co-existence of other complementary arrangements which involve other structure types. The structures are there and must be explained! We encourage theoreticians to elaborate new models which can help us to understand all these features.

Our conclusion is that an important procedure for gaining insight into crystal structures is not to restrict the contemplation to anions and cations in their conventional oxidation states, but also to contemplate how selected pairs of atoms might accommodate their valence electrons to produce pseudo-structures characteristic of the elements of Group 14. If this is the driving force, the conventional oxidation states assigned to cations and anions lose some of their usefulness in accounting for crystal structures.

## Figures and Tables

**Table 1:** List of stuffed anti-bixbyite-type compounds Those crystallizing in the 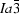 space group preserve the space group of bixbyite itself. The stuffing elements occupy the 16*c* site of 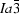. In Li_6_[Li,V]N_4_, crystallizing in the subgroup 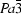, the lower symmetry preserves the unit-cell dimensions but separates both V and Li atoms into two distinct 8*c* sites.

Compound	Stuffing cation	Space group	References
Li_3_AlN_2_	Al	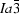	Juza & Hund (1948 ▶)
Li_3_GaN_2_	Ga	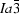	Juza & Hund (1948 ▶)
Li_3_ScN_2_	Sc	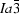	Niewa *et al.* (2003 ▶)
Li_3_[Ge_0.67_Li_0.33_]N_2_	Ge, Li	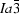	Juza *et al.* (1953 ▶)
Li_3_[Si_0.67_Li_0.33_]N_2_	Si, Li	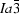	Juza *et al.* (1953 ▶)
Li_3_[Ti_0.67_Li_0.33_]N_2_	Ti, Li	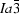	Juza *et al.* (1953 ▶)
Li_6_[MnLi]N_4_	Mn, Li	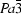	Niewa *et al.* (2001 ▶)
Li_6_[VLi]N_4_	V, Li	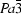	Niewa & Kniep (2001 ▶)
Li_6_[NbLi]N_4_	Nb, Li	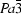	Vennos & DiSalvo (1992 ▶)
Li_6_[TaLi]N_4_	Ta, Li	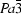	Wachsmann & Jakobs (1992 ▶)

**Table 2:** Possible electron redistributions for species relevant to the present work The Zintl–Klemm notation describes the electron redistribution, so that, in the overall electron count for the elements concerned, the superscript −1 (rather than 1−), for example, represents an *excess* electron on that atom; the superscript +1 represents a one-electron loss. Thus, Li^+1^ is (Ψ-He), Al^−1^ and N^−1^ are (Ψ-Si) and (Ψ-O), respectively, and Al^+3^ is (Ψ-Ne).

Electron donation				Electron acceptance		
3	2	1	Atom	1	2	3
N^+3^ = (Ψ-Be)	N^+2^ = (Ψ-B)	N^+1^ = (Ψ-C)	N^0^	N^−1^ = (Ψ-O)	N^−2^ = (Ψ-F)	N^−3^ = (Ψ-Ne)
	Li^+2^ = (Ψ-H)	Li^+1^ = (Ψ-He)	Li^0^	Li^−1^ = (Ψ-Be)	Li^−2^ = (Ψ-B)	Li^−3^ = (Ψ-C)
Al^+3^ = (Ψ-Ne)	Al^+2^ = (Ψ-Na)	Al^+1^ = (Ψ-Mg)	Al^0^	Al^−1^ = (Ψ-Si)	Al^−2^ = (Ψ-P)	Al^−3^ = (Ψ-S)
V^+3^ = (Ψ-Ca)	V^+2^ = (Ψ-Sc)	V^+1^ = (Ψ-Ti)	V^0^	V^−1^ = (Ψ-Cr)	V^−2^ = (Ψ-Mn)	V^−3^ = (Ψ-Fe)

**Table 3:** Summary of various electron redistributions discussed in the text

Case	Normal compound	Zintl–Klemm notation	Pseudo-phase
	Atomic species	Electron redistribution	Phase description
	Phase description		
1	Li_3_AlN_2_	(Li^+1^)_3_[Al^−1^(N^−1^)_2_]	(Ψ-He_3_SiO_2_)
	(Li^0^)_3_Al^0^(N^0^)_2_	(Ψ-He)_3_(Ψ-Si)(Ψ-O)_2_	‘Si-stuffed He_3_O_2_ anti-bixbyite’
	‘Al-stuffed Li_3_N_2_ anti-bixbyite’		
2	Li_3_AlN_2_	(Li^−1^)_3_Al^+3^(N^0^)_2_	(Ψ-Be_3_NeN_2_)
	(Li^0^)_3_Al^0^(N^0^)_2_	(Ψ-Be)_3_(Ψ-Ne)(N^0^)_2_	‘Ne-stuffed Be_3_N_2_ anti-bixbyite’
3	Li_3_AlN_2_	[Li_3_Al]^−2^(N^+1^)_2_	(Ψ-Li_3_PC_2_) ≡ (Ψ-Mg_4_Si_2_)
	(Li^0^)_3_Al^0^(N^0^)_2_	(Ψ-Li_3_P)(Ψ-C)_2_	‘P-stuffed Li_3_C_2_ anti-bixbyite’
4	Li_3_AlN_2_	(Li^+1^)_2_[Li^−2^Al^0^(N^0^)_2_]	(Ψ-He)_2_(Ψ-BAlN_2_)
	(Li^0^)_3_Al^0^(N^0^)_2_	(Ψ-He)_2_[(Ψ-B)Al^0^](N^0^)_2_	‘Blende’
5	Li_3_(Ge_0.667_Li_0.333_)N_2_	(Li^+1^)_3_[(Li^−3^)_0.333_(Ge^0^)_0.667_](N^−1^)_2_	(Ψ-He_3_Si_0.333_Ge_0.667_O_2_)
	(Li^0^)_3_[(Ge^0^)_0.667_(Li^0^)_0.333_](N^0^)	(Ψ-He)_3_[(Ψ-C)_0.333_Ge_0.667_](Ψ-O)_2_	‘Si/Ge-stuffed He_3_O_2_ anti-bixbyite’
	‘Ge/Li-stuffed Li_3_N_2_ anti-bixbyite’		
6	Li_6_[V^V^Li]N_4_	(Li^+1^)_3_ [(Li^−3^)_0.5_(*M*^+1^)_0.5_](N^−1^)_2_	Ψ-He_3_(C_0.5_Ti_0.5_)O_2_
	(Li^0^)_6_[V^0^Li^0^](N^0^)_4_	(Ψ-He)_3_[(Ψ-C_0.5_Ti_0.5_)](Ψ-O)_2_	‘Ti/C-stuffed He_3_O_2_ anti-bixbyite’
	V/Li-stuffed Li_3_N_2_ anti-bixbyite		

**Table 4:** CD representations for cases 1–3

		Anti-CD representation		
				Overall formula
Case	Zintl–Klemm	Core tetrahedron	Peripheral tetrahedron	Pseudo-compound
1	(Li^+1^)_3_Al^−1^(N^−1^)_2_	[Al^−1^(N^−1^)_4/8_]^−1.5^	[Li^+^(N^−1^)_4/8_]^+0.5^	[Al^−1^(N^−1^)_4/8_]^−1.5^{[Li^+1^(N^−1^)_4/8_]^+0.5^}_6/2_
				(Ψ-He_3_SiO_2_)
2	(Li^−1^)_3_Al^+3^(N^0^)_2_	[Al^+3^(N^0^)_4/8_]^+3^	[Li^−1^(N^0^)_4/8_]^−1^	[Al^+3^(N^0^)_4/8_]^+3^{[Li^−1^(N^0^)_4/8_]^−1^}_6/2_
				(Ψ-Be_3_NeN_2_)
3	[Li_3_Al]^−2^(N^+1^)_2_	[Al^−2^(N^+1^)_4/8_]^−1.5^	[Li^0^(N^+1^)_4/8_]^+0.5^	[Al^−2^(N^+1^)_4/8_]^−1.5^{[Li^0^(N^+1^)_4/8_]_6/2_}^+1.5^
				(Ψ-Li_3_PC_2_),

**Table:** 

Core tetrahedron	Peripheral tetrahedron	Overall CD formula
Case 5	Zintl–Klemm distribution	(Li^+1^)_3_(Li^−3^)_0.333_(Ge^0^)_0.667_(N^−1^)_2_
[(Li^−3^)_0.333_(Ge^0^)_0.667_(N^−1^)_4/8_]^−1.5^	[Li^+1^(N^−1^)_4/8_]^+0.5^	[(Li^−3^)_0.333_(Ge^0^)_0.667_(N^−1^)_4/8_]^−1.5^{[Li^+1^(N^−1^)_4/8_]^+0.5^}_6/2_

**Table:** 

Core tetrahedron	Peripheral tetrahedron	Overall CD formula
Case 6	Zintl–Klemm distribution	(Li^+1^)_6_[(Li^−3^)(*M*^+1^)](N^−1^)_4_
(1) [Li^−3^(N^−1^)_4/8_]^−3.5^	[Li^+1^(N^−1^)_4/8_]^+0.5^	[Li^−3^(N^−1^)_4/8_]^−3.5^{[Li^+1^(N^−1^)_4/8_]^+0.5^}_6/2_ (net charge −2)
(2) [*M*^+1^(N^−1^)_4/8_]^+0.5^	[(Li^+1^)(N^−1^)_4/8_]^+0.5^	[*M*^+1^(N^−1^)_4/8_]^+0.5^{[(Li^+1^)(N^−1^)_4/8_]^+0.5^}_6/2_ (net charge +2)
